# Efficacy of a Novel Topical Combination of Fipronil 9.8% and (S)-Methoprene 8.8% against Ticks and Fleas in Naturally Infested Dogs

**DOI:** 10.1155/2016/7174685

**Published:** 2016-04-17

**Authors:** Ayyanampakkam Pandurangan Nambi, Badal Rathi, Kavitha S, Ghanshyam Dudhatra, Hamsa S. Yamini, Abid Ali Bhat

**Affiliations:** ^1^Department of Veterinary Medicine, Ethics & Jurisprudence, Madras Veterinary College, Chennai, Tamil Nadu 600007, India; ^2^SAVAVET, Sava Healthcare Limited, Viman Nagar, Pune, Maharashtra 411014, India

## Abstract

The efficacy of a novel topical combination of fipronil 9.8% (w/v) and (S)-methoprene 8.8% (w/v) (Fiprofort® Plus) was tested against ticks and fleas in naturally infested dogs. A total of fifty dogs were allocated in the study with ticks infestation (*n* = 35) and fleas infestation (*n* = 15). On day 0, thirty-five tick and fifteen flea infested dogs received the test formulation, a combination of fipronil 9.8% (w/v) and (S)-methoprene 8.8% (w/v) spot-on solution. Ticks and flea counts were taken on days 0 (pretreatment) and 3, 7, 14, 21, 28, and 35 after treatment. Blood samples were collected for evaluation of haematological parameters on days 0 (pretreatment) and 7, 21, and 35 after treatment. All the adult ticks and fleas collected were identified as* Rhipicephalus sanguineus* and* Ctenocephalides felis*, respectively. The efficacy of spot-on formulation against ticks was 34.00% (day 3), 53.14% (day 7), 62.71% (day 14), 65.48% (day 21), 59.80% (day 28), and 58.82% (day 35), whereas against fleas it was 38.00% (day 3), 64.34% (day 7), 89.67% (day 14), 95.40% (day 21), 100.00% (day 28), and 100.00% (day 35). Haematological parameters for ticks and fleas infested dogs were statistically nonsignificant as compared to control. The combination of fipronil and (S)-methoprene eliminated the existing ticks and fleas infestation and prevented the dogs from flea and tick infestation for four weeks.

## 1. Introduction

Ticks and fleas infestations represent an important problem for dogs and their owners practically worldwide. The brown dog tick,* Rhipicephalus sanguineus*, is a three-host tick able to complete each stage on dogs, even indoors [1, 2], and acts as most important vector for several pathogens which cause severe canine diseases [3–6]. The dog flea,* Ctenocephalides felis*, is the most prevalent insect, and its population is mostly constituted by immature stages that infest dogs [7–9]. Fleas are also vectors of several pathogens [4, 10–12].

Adequate control of ticks and fleas infestation helps in relieving the immediate distress caused to their hosts, such as itching, skin lesions, and blood loss, and may also prevent the direct effects such as tick-induced paralysis and flea allergy dermatitis. A reduction in parasite numbers may have an effect on the prevalence as well as transmission of vector-borne diseases [13].

Several combinations with acaricidal or insecticidal properties and which are appropriate and safe for treatment of dogs have been formulated for application either orally, parentally, topically, or as medicated collars. Depending on the active ingredients of the chemicals or combinations of chemicals, they are reported to be effective against fleas or ticks, or both [14–16].

The objective of the study presented in this paper was to investigate the efficacy and safety of topical combination of fipronil 9.8% and (S)-methoprene 8.8% against ticks and fleas in naturally infested dogs in Indian environmental conditions.

## 2. Materials and Methods

### 2.1. Animals

Fifty dogs (*n* = 35 for ticks and *n* = 15 for fleas) of either sex of different breeds weighing between 10 and 48 kg and aged more than 6 months were included in the study. The dogs were screened for presence of external parasites and few representative specimens from infested dogs were collected for laboratory identification. Before treatment, the good health of each dog was confirmed by a physical examination. Dogs were handled with due regard to their welfare. Water was available* ad libitum* and an adequate amount of a commercial dog food towards their maintenance was provided daily. To detect the presence or absence of any treatment-related or treatment-unrelated health abnormality or adverse event, health observations were conducted daily throughout the study period. The study protocol was reviewed and approved by the Madras Veterinary College Institutional Ethics Committee, Tamilnadu Veterinary and Animal Sciences University, Chennai, India.

### 2.2. Treatment

On day 0, dogs received Fiprofort Plus (SAVAVET, Sava Healthcare Limited, Pune, India) containing fipronil and (S)-methoprene combination spot-on of 0.67 mL (for dogs weighing between 1 and 10 kg), 1.34 mL (for dogs weighing between 10.1 and 20 kg), 2.68 mL (for dogs weighing between 20.1 and 40 kg), and 4.02 mL (for dogs weighing over 40 kg) according to their body weight. The treatments were applied directly onto the skin, after parting the hair, in one spot on the midline of the neck between the base of the skull and the shoulder blades.

### 2.3. Intensity of Ticks and Fleas Infestation

The intensity of ticks and fleas in naturally infested dogs was noted on days 0, 3, 5, 7, 14, 21, 28, and 35, respectively. The intensity of tick infestation was expressed as number of ticks per unit area of 4 square inches. The intensity of flea infestation was noted by passing the flea comb five times from head to tail and number of fleas trapped in the comb during the process was counted and expressed as intensity of fleas on the body.

Few specimens of ticks and fleas from representative dogs included in the study were collected and processed in the laboratory for preparation of permanent mounts as per the method described by Soulsby [17] with few modifications. The specimens were initially treated overnight with 5% potassium hydroxide followed by dehydration in ascending grades of alcohol and clearing in carboxyl before mounting on glass slide with Canada balsam.

### 2.4. Collection of Blood Samples

The blood samples were collected for haematological profile of each dog on days 0, 7, 21, and 35, respectively. Haematological parameters included haemoglobin, Packed Cell Volume (PCV), Red Blood Cell Counts (RBC), White Blood Cell Counts (WBC), and Platelet Counts.

### 2.5. Laboratory Analysis

The ticks and fleas collected from representative cases were processed in the laboratory of Department of Veterinary Medicine, Madras Veterinary College, Chennai, for identification of the ectoparasites. Blood samples for haematological profile were also conducted in the same laboratory.

### 2.6. Statistical Analysis

Efficacy against ticks and fleas was calculated for the treated groups at each assessment day in accordance with WAAVP guidelines, using Abbott's formula [18]:

(1) $$\begin{array}{c} \text{Efficacy}\left(\;\%\;\right)=100\times \frac{M_{c}-M_{t}}{M_{c}}, \end{array}$$

where *M*_*c*_ is the mean of live ticks/fleas on the control group and *M*_*t*_ is the mean of live ticks/fleas on the treatment groups.

The groups were compared by one-way ANOVA statistical analysis using SPSS software for haematological parameters.

## 3. Results

None of the study dogs showed signs of irritation or skin lesions after application of Fiprofort Plus spot-on formulation.

### 3.1. Efficacy of Fiprofort Plus Spot-On against Tick Infestation in Dogs

**Figure 1:**
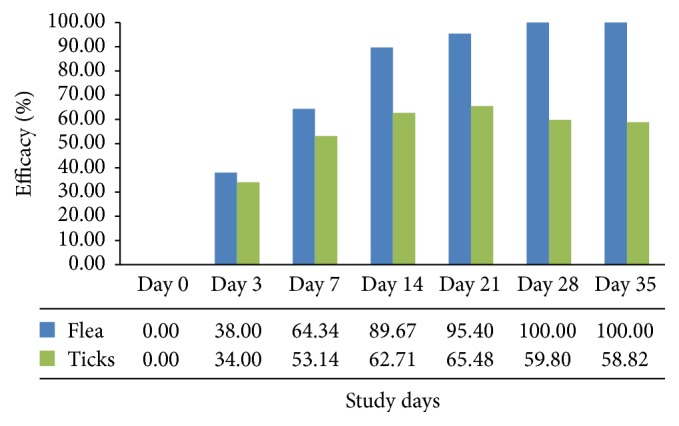
Efficacy (%) of Fiprofort Plus spot-on application against ticks and fleas during 35 days of the study.

Efficacy and speed of kill of Fiprofort Plus spot-on against ticks during the study are depicted in Table 1 and Figure 1.

On day 0, the mean tick count was 195. On day 3, the mean tick count was 128 resulting in an overall decrease of preexisting population of 34.00% (Table 1) as compared to day 0. The mean tick count was recorded to be 67 on day 21 with overall decrease of 65.42% as compared to day 0, which stands for the highest reduction during the trial period. The speed of kill on days 3 and 21 was observed to be 34.00% and 65.42%, respectively; thereafter, reversal trend was seen as mean tick load started increasing after day 21.

In this study, overall efficacy was highly significant on day 21 (65.48%) followed by day 14 (62.71%), with minimal efficacy on day 3 (34.00%). The persistence of efficacy of 58.82% was recorded on day 35, which was the termination day of the trial period.

### 3.2. Efficacy of Fiprofort Plus Spot-On against Flea Infestation in Dogs

Efficacy and speed of kill of Fiprofort Plus spot-on against fleas during the study are depicted in Table 2 and Figure 1.

On day 0, the mean flea count was 37. On day 3, the mean flea count was 23 resulting in an overall decrease of preexisting population of 38.00% (Table 2) as compared to day 0. The mean flea count was recorded to be 2 on day 21 with overall decrease of 95.40% as compared to day 0. The speed of kill on days 14 and 21 was observed to be 89.67% and 95.40%, respectively.

In this study, overall efficacy was strongest on days 28 and 35 (100% each) of the trial period. 100% efficacy against flea was noticed at the end of the trial, indicating persistence of the efficacy of the Fiprofort Plus beyond trial period. Speed of kill of Fiprofort Plus was high against fleas as compared to ticks.

### 3.3. Haematological Profile of Fiprofort Plus Spot-On against Ticks and Flea Infestation in Dogs

Mean ± SE values for various haematological parameters calculated in both tick and fleas groups were given in Tables 3 and 4, respectively. No significant difference was observed before and after treatment in all the days against ticks and fleas infested dogs.

## 4. Discussion

In the present study, the spot-on formulation containing fipronil and (S)-methoprene showed variable efficacy against ticks (34.00 to 65.48%) and high efficacy against fleas (100%) for a month. The results demonstrated the overall high level of efficacy of spot-on formulation against ticks and fleas in naturally infested dogs, as previously reported for fipronil and (S)-methoprene [13, 19–24]. Also, haematological parameters showed no significant difference against ticks and fleas infested dogs as compared to positive control.

The combination of fipronil and (S)-methoprene in the Fiprofort Plus offers a wide spectrum of efficacy against the fleas and ticks in naturally infested dogs. The control of ticks and fleas is important for prevention of zoonotic diseases. The need for such treatments will be determined by the veterinarian, based on diagnosis and risk assessment according to the region and the dog's environment. Using a novel approach for dogs presenting ticks and flea infestations, this combination product will be a distinct advantage. The safety and efficacy of a combination spot-on product have been demonstrated in the present study under Indian environmental conditions and its ease of application may improve owner compliance.

## Figures and Tables

**Table 1:** Efficacy and speed of kill of Fiprofort Plus spot-on against ticks during 35 days of the study.

Study day	Count		Percentage		% efficacy
	Live	Dead	Live	Dead	
0	195	0	100.00	0.00	0.00
3	128	66	66.00	34.00	34.00
7	91	103	46.94	53.06	53.14
14	73	122	37.36	62.64	62.71
21	67	127	34.58	65.42	65.48
28	78	116	40.27	59.73	59.80
35	80	114	41.25	58.75	58.82

Efficacy (%) = 100 × (*M*_*c*_ − *M*_*t*_)/*M*_*c*_, where *M*_*c*_ is the mean of live ticks on the control group and *M*_*t*_ is the mean of live ticks on the treatment groups.

Day 0 is control (untreated).

**Table 2:** Efficacy and speed of kill of Fiprofort Plus spot-on against fleas during 35 days of the study.

Study day	Count		Percentage		% efficacy
	Live	Dead	Live	Dead	
0	37	0	100.00	0.00	0.00
3	23	23.56	62.00	38.00	38.00
7	13	22.94	35.66	64.34	64.34
14	4	9.26	10.33	89.67	89.67
21	2	4.39	4.60	95.40	95.40
28	0	0	0.00	100.00	100.00
35	0	0	0.00	100.00	100.00

Efficacy (%) = 100 × (*M*_*c*_ − *M*_*t*_)/*M*_*c*_, where *M*_*c*_ is the mean of live fleas on the control group and *M*_*t*_ is the mean of live fleas on the treatment groups.

Day 0 is control (untreated).

**Table 3:** Haematological profiles of tick infested dogs before and after Fiprofort Plus application.

Study day	Haemoglobin (g/dL)	PCV (%)	RBC (×10^6^/cmm)	WBC (×10^3^/cmm)	Platelet (×10^5^/cmm)
0	13.31 ± 0.28	39.44 ± 0.92	5.69 ± 0.15	13.06 ± 2.6	2.26 ± 0.92
7	13.21 ± 0.29	38.76 ± 0.97	5.62 ± 0.14	13.39 ± 3.6	2.49 ± 1.57
21	13.32 ± 0.29	39.06 ± 0.93	5.61 ± 0.15	15.68 ± 3.5	2.39 ± 1.24
35	13.1 ± 0.44	39.04 ± 0.92	5.64 ± 0.14	15.66 ± 1.5	2.38 ± 0.96

Significance	0.682^NS^	0.65^NS^	0.64^NS^	0.583^NS^	0.214^NS^

NS: statistically nonsignificant (*P* > 0.05).

Day 0 is control (untreated).

**Table 4:** Haematological profiles of flea infested dogs before and after Fiprofort Plus application.

Study day	Haemoglobin (g/dL)	PCV (%)	RBC (×10^6^/cmm)	WBC (×10^3^/cmm)	Platelet (×10^5^/cmm)
0	12.81 ± 0.16	36.91 ± 0.75	5.48 ± 0.14	15.52 ± 5.18	2.55 ± 0.15
7	12.47 ± 0.23	34.59 ± 0.84	5.20 ± 0.12	10.13 ± 0.45	3.06 ± 0.35
21	12.79 ± 0.25	36.48 ± 0.67	5.23 ± 0.11	8.87 ± 0.42	2.73 ± 0.33
35	13.05 ± 0.21	36.91 ± 1.34	5.34 ± 0.12	11.92 ± 1.58	3.03 ± 0.28

Significance	0.100^NS^	0.120^NS^	0.140^NS^	0.347^NS^	0.215^NS^

NS: statistically nonsignificant (*P* > 0.05).

Day 0 is control (untreated).
